# Clinical and Pathological Tools for Predicting Recurrence and/or Metastasis in Patients with Pheochromocytoma and Paraganglioma

**DOI:** 10.3390/biomedicines10081813

**Published:** 2022-07-28

**Authors:** Chiara Bima, Fabio Bioletto, Chiara Lopez, Martina Bollati, Stefano Arata, Matteo Procopio, Iacopo Gesmundo, Ezio Ghigo, Mauro Maccario, Mirko Parasiliti-Caprino

**Affiliations:** Endocrinology, Diabetes and Metabolism, Department of Medical Sciences, University of Turin, 10126 Turin, Italy; chiara.bima@unito.it (C.B.); fabio.bioletto@unito.it (F.B.); chiara.lopez@fastwebnet.it (C.L.); bollati.martina@gmail.com (M.B.); stefano16196@gmail.com (S.A.); mprocopio35@gmail.com (M.P.); iacopo.gesmundo@unito.it (I.G.); ezio.ghigo@unito.it (E.G.); mauro.maccario@unito.it (M.M.)

**Keywords:** pheochromocytoma, paraganglioma, malignancy, recurrence, prognostic factors, predictive model

## Abstract

Pheochromocytomas and paragangliomas are endocrine tumors belonging to the family of neural crest cell-derived neoplasms. They have an extremely variable clinical course, characterized by a non-negligible percentage of relapse and/or metastasis after radical surgery. To date, there are no reliable methods to predict the metastatic potential of these neoplasms, despite several clinical, molecular, and histopathological factors that have been extensively studied in the literature as predictors of the recurrence and/or metastasis in these neoplasms with different performances and results. In this review, we aimed to discuss and analyze the most important clinical and histopathological tools for predicting recurrence risk in patients affected by pheochromocytomas or paragangliomas. Thus, we compared the main available predictive models, exploring their applications in stratifying patients’ risks. In conclusion, we underlined the importance of simple and validated tools to better define disease aggressiveness and establish tailored patients’ treatments and follow-ups.

## 1. Introduction

Pheochromocytomas and paragangliomas (PPGLs) are rare neuroendocrine tumors (NETs) derived from adrenomedullary chromaffin cells and from the autonomic paraganglia, respectively. Pheochromocytomas (PCCs) represent about 80–85% of chromaffin-cell neoplasms, whereas paragangliomas (PGLs) account for the remaining 15–20%. PCCs and sympathetic PGLs are typically catecholamine-producing tumors. Instead, parasympathetic PGLs are often non-functioning or dopamine-producing neoplasms. PPGL are characterized by an annual incidence of approximately two to eight cases per million inhabitants with a prevalence of 0.2–0.6% in hypertensive patients. About 5% of patients with adrenal incidentaloma are affected by PCC [1]. Furthermore, about 30–40% of patients with PPGLs have hereditary predispositions [2]. The clinical signs and symptoms of PPGLs are very variable and non-specific; thus, the consequent delayed or missed diagnosis could be fatal or lead to significant complications and adverse outcomes [3,4]. PPGLs are also very heterogeneous in the clinical course with a variable prognosis concerning the development of metastases. Despite most of these neoplasms are radically cured by surgery, all PPGLs have potentially metastatic properties, and no reliable clinical, histopathological, or biochemical predictors are established to determine with certainty whether a PPGL could be potentially metastatic [5,6,7]. In the recent position statement of the European Society of Hypertension, the expert panel discussed the importance of defining a reliable prediction of metastatic disease and the linking to the underlying genetic background to facilitate personalized follow-up [8]. Although many studies have examined the potential prognostic role of various clinical, biochemical, genetic, and histopathological features, there is not a single feature that can be used alone to reliably predict tumor recurrence and, therefore, guide clinical practice [9,10,11]. Therefore, in this review, we aim to discuss and analyze the most important clinical and pathological tools for predicting recurrence and/or metastatic risk in patients with PPGLs. 

## 2. Metastatic Disease and/or Recurrence in PPGLs: Background

In the latest World Health Organization (WHO) Classification for Endocrine Tumors [5], the discrimination between benign versus malignant PPGLs has been removed; thus, the neoplasms are now classified as metastatic or not, according to the presence of metastases in non-chromaffin tissue, such as lymph nodes or other distant sites. Approximately 10% of PCCs (5–26% according to several studies) and up to 35% of PGLs are metastatic [12]. However, their risk of recurrence is even higher, as they may recur not only with the development of metastases but also because of local recurrence, defined as the development of the disease in the primary tumor site or in other chromaffin-derived tissues, such as the contralateral adrenal gland [3,13]. Complete resection of the primary tumor can be curative for most patients, but recurrence risk is still not negligible, neither in the short nor in the long term [1,3,13,14,15]. 

A recent systematic review and meta-analysis estimated a recurrence rate of around 1% every year [3]. In this meta-analysis of 42 studies published from 1980 to 2012, Amar et al. [3] suggested a lower risk of PPGL recurrence compared to previous literature evidence (approximately 5% per five years of follow-up, variable from 1 to 34%). However, the authors also underlined the limitations of the available evidence, according to the heterogeneity of the included studies and the lack of standardized follow-up. Thus, they could not derive a firm conclusion on this topic. 

In addition, the progression of the metastatic disease seems to be very heterogeneous. Goffredo et al. [16] conducted a retrospective study on 508 patients affected by PPGL drawn from 18 state registries (from 1988 to 2009) and documented that the overall and disease-specific survival was extremely variable, resulting generally lower for PCCs than PGLs. 

Recently, a retrospective study by Hamidi et al. [17], conducted on 272 patients with metastatic disease (21–22% in stage IV at diagnosis), highlighted that the clinical course of the metastatic disease was extremely variable, and documented an overall and disease-specific survivals of 24.6 and 33.7 years, respectively. The authors also showed that poorer prognosis was related to male gender, larger primary tumor size, older age at diagnosis, elevated dopamine levels, synchronous metastases, and non-radical surgery. 

In another meta-analysis, Hamidi et al. [18] also described the outcomes of metastatic PPGLs. The main results suggested low mortality rates of patients with metastatic PPGLs with worse prognoses in the cases of male gender and synchronous metastases. However, the authors highlighted scarce quality of the available evidence due to important referral bias and heterogeneity of the studies that often include patients not radically cured, defining only the risk of metastatic disease. Therefore, they concluded that further research is needed to obtain prognostic information in this field.

## 3. Clinical Predictors

The rate of metastatic disease is extremely variable. Although some features, including larger tumor size (especially more than 5 cm), extra-adrenal primary tumor site, younger age at diagnosis, or elevated levels of plasma 3-methoxytyramine (3-MT) [1] could guide clinical practice to establish the risk of developing metastasis, the presence of mutations in the succinate dehydrogenase type B (*SDHB*) gene is generally considered as the strongest single risk factor associated with a significant risk of metastatic disease, leading to metastases in 40% or more of patients [1,19,20]. However, in a recent retrospective study conducted on a large series of 169 metastatic PPGLs, the authors did not confirm *SDHB* mutation as a major prognostic parameter in metastatic disease, suggesting the potential role of other molecular events in tumor progression [4]. The available literature concerning clinical features, secretory phenotype, and tumor morphological characteristics is controversial as well. Li M. et al. [21] conducted a retrospective study on 249 patients (43 affected by metastatic PPGLs and 206 without metastatic PPGLs) to analyze the clinical features of metastatic disease. They did not find any differences in signs and symptoms between the two groups, suggesting that no clinical pattern could be helpful in predicting metastatic properties. In the following paragraphs, we analyzed the main clinical tools that can be useful as potentially prognostic factors of aggressive disease.

### 3.1. Age at Diagnosis

Younger age at diagnosis is typically associated with more aggressive disease [14,22,23,24], mainly due to the correlation with hereditary syndromes. In fact, in pediatric patients, most of these neoplasms are represented by extra-adrenal PGLs, typically related to hereditary background [25].

### 3.2. Biochemical Markers

The literature data showed that the recurrence risk of PPGLs is associated with the secretory phenotype [26], reflected by higher norepinephrine levels and lower epinephrine/epinephrine + norepinephrine ratio in metastatic disease. The downregulation of the phenylethanolamine *N*-methyltransferase (PNMT) could explain the higher levels of norepinephrine in metastatic disease or Von Hippel-Lindau (VHL)-related chromaffin tumors [27]. Concerning this issue, Ayala-Ramirez et al. [10] demonstrated that after normalizing the urinary excretion of catecholamines per unit of tumor volume, metastatic PPGLs had lower epinephrine levels compared to the other metabolites. Additionally, Eisenhofer et al. [28] conducted a study on 365 PPGLs patients and demonstrated higher norepinephrine, normetanephrine, and 3-MT levels in metastatic tumors, underlining that plasma 3-MT was the most accurate biomarker for discrimination of metastatic disease. Among biochemical markers, 3-MT is a dopamine metabolite that has been extensively studied in the literature as a predictor of recurrence [29,30], resulting as useful when measured in plasma, not only as a predictor of metastatic disease but also as an independent predictor of survival among patients with PPGLs [31]. The prognostic value of chromogranin A (CgA) was also explored in the literature. Rao et al. [32] observed that CgA was significantly different in benign versus metastatic PCCs, and they also found a correlation between CaA levels and recurrence risk. 

### 3.3. Tumor Site and Size

Tumor size was extensively associated with recurrence risk, as shown by Park et al. [33], Ayala-Ramirez et al. [10], Press et al. [34], Amar et al. [35], Feng et al. [36], Eisenhofer et al. [28], Assadipour et al. [37], De Wailly et al. [38], and our group [14] who observed a strong correlation between lesion size and metastatic disease or patient survival. Concerning bilateral localization of PCCs, literature data are discordant. Some studies, as underlined by Park et al. [33] and John et al. [39], showed that bilateral disease was not associated with metastatic potential, while other ones demonstrated a significant correlation, as observed by Feng et al. [36]. The evaluation of this variable as a predictor of recurrence could be influenced by genetic background, as many bilateral localizations are related to genetic syndromes, as shown by our group [14]. Regarding the extra-adrenal localization, the available evidence is still a subject of debate. In fact, some authors found a positive correlation with recurrence risk. John et al. [39] discovered that extra-adrenal tumor location was related to metastatic disease in up to 36% of cases. Furthermore, Ayala-Ramirez et al. [10] described a higher risk of metastasis in PGLs than in PCCs, about 4.5 times higher. Conversely, Cho et al. [22] did not find a correlation between the localization of the tumor and the prognosis of PPGLs, and Goffredo et al. [16] demonstrated a higher incidence of recurrence in the case of adrenal location. 

### 3.4. Functional Imaging

Chromaffin tumors with metastatic potential are less differentiated and could have a lower ability to uptake PPGL-specific tracers, but to our knowledge, the capability of functional-imaging techniques in the prediction of aggressive disease has not been defined. In fact, the Nuclear Medicine Guidelines [40] and the consensus statement of the European Society of Hypertension [8] proposed a personalized approach to the use of functional-imaging modalities only in the staging of recurrent or metastatic disease, according to different clinical scenarios and genetic backgrounds. The recent available literature suggested in metastatic PPGLs a preferential role for some radiopharmaceuticals, such as ^18^F-fluoro-2-deoxy-D-glucose (^18^F-FDG) [41], ^18^F-fluorodihydroxyphenylalanine (^18^F-FDOPA) [42], and ^68^Gallium-labelled somatostatin analogue (^68^Ga-SSA) [43,44,45]. However, the clinical relevance and implications of functional imaging in PPGL management is still being debated [14] because the role of functional imaging is well defined concerning diagnosis or staging but not as a predictor of recurrence or metastatic disease.

### 3.5. Genetic Background

A genetic germline cause can be identified in approximately 30–40% of PPGLs [2]. The most common familial or syndromic PPGLs are related to germline mutations in genes encoding the subunits of SDH (*SDHD*; *SDHAF2*; *SDHC*; *SDHB*; *SDHA*) (15–20%), the Von Hippel-Lindau (*VHL*) gene (9%), the *RET* proto-oncogene, causing multiple endocrine neoplasia-2 (MEN2) syndrome (5%), and the neurofibromatosis type 1 (*NF-1*) gene (2%). Less frequent familial forms (<1–2%) are caused by mutations in the transmembrane protein 127 (*TMEM127*), MYC-associated factor X (*MAX*), fumarate hydratase (*FH*), multiple endocrine neoplasia type 1 (*MEN1*), egg-laying-defective nine (*egl-9*) family hypoxia-inducible factor 1 gene (*EGLN1*), egl-9 family hypoxia-inducible factor 2 (*EGLN2*), malate dehydrogenase 2 (*MDH2*), kinesin family member 1B (*KIF1B*) genes, solute carrier family 25 Member 11 (*SLC25A11*), and dihydrolipoamide S-succinyl transferase [46,47]. The main germline and somatic mutations in more than 20 PPGL driver genes are divided into three main molecular clusters: pseudohypoxia cluster 1 (1A and 1B), kinase-signaling (cluster 2), and Wnt-signaling (cluster 3) [48]. The cluster 1 group tumors are characterized by mutations in genes involved in the Krebs cycle that leads to HIF1A or HIF1B stabilization and consequently, to a condition of pseudohypoxia, which determines increased angiogenesis and elevated cell proliferation. These neoplasms are also showing a typical pattern of hypermetylation that leads to deregulation of genes involved in neuroendocrine differentiation or in the epithelial–mesenchymal transition process with consequently higher metastatic properties [11]. Recent studies also reported that immortalization mechanisms concerning telomere dysfunction, also contribute to PPGL progression. In fact, the activation of the telomerase gene, *TERT*, *ATRX* loss of function mutations, or *NOP10* overexpression have been described in association with adverse prognosis in PPGL [49]. 

In familial forms, PPGLs are typically characterized by bilateral or extra-adrenal location, association with multiple other neoplasms, and elevated recurrence risk [8]. The rate of metastatic disease varies greatly depending on the genetic background [1,2,9,28,35] with a low rate in cases of *RET* and *SDHD* mutations and in VHL disease, a rate of about 12% in NF-1, and up to 30–70% in cases of *SDHB* mutations [4,50,51]. Therefore, the assessment of genetic background is of crucial importance because a significant percentage of apparently sporadic tumors may also be caused by a germline mutation [52]. Endocrine Society [1] and European Society of Endocrinology Guidelines [13] currently recommend considering genetic testing in all patients with PPGLs because modern genetic counselling is a fundamental way to ensure familial case detection and tailored treatment.

The main studies cited in the text analyzing clinical predictors of metastatic disease/recurrence are summarized in Table 1.

## 4. Histopathological Scores

Some histological features, such as tumor necrosis, mitoses over three per ten high-power fields (HPF), high cellularity, capsular invasion, and vascular or adipose tissue invasions have been proposed as predictive markers of more aggressive tumors [38]. However, the true predictive performance of many of these parameters, such as mitotic activity, cellular atypia, or vascular invasion, is quite scarce. Similarly, the extension of local invasion to adjacent tissues also does not necessarily mean a higher risk for the metastatic evolution [53]. The proliferation marker, Ki67, has also been used to predict the metastatic behavior of PPGLs. However, the Ki67 proliferative index is characterized by high specificity compared to low sensitivity because almost half of malignant PCCs are characterized by a Ki-67 index <2–3%, and this condition could be related to the limited number of cells engaged in the Ki-67 expressing phase of the cell cycle [38,54]. In addition, several molecular markers, such as SDHB, MAML3, SNAIL, hTERT, HSP90, STAT3, HuR, COX-2, VEGF, HIF1alpha, and secretogranin, have also been related to metastatic PPGLs [55]. The loss of expression of SDHB in the immunohistochemical studies demonstrated the presence of an *SDH* germline mutation and an increased risk of metastases development [56]. Based on these data, different scoring systems have been proposed to estimate the metastatic risk of these neoplasms.

### 4.1. Pheochromocytoma of the Adrenal Gland Scaled Score (PASS)

The first scoring system proposed is the pheochromocytoma of the adrenal gland scaled score (PASS), elaborated through a retrospective study conducted on a cohort of 100 PCC cases [57]. This multiparametric-scoring system is based on 12 specific histological features that are more frequently identified in metastatic PCCs, as summarized in Table 2. The score related to these parameters were called PASS, and it correlated with metastatic properties in the cases of tumors with a PASS equal or greater than four. Several studies explored the association of PASS with recurrence and/or metastatic behavior to validate the scoring system [14,23,38,57,58,59]. Strong et al. [24] conducted a study on 51 PCC2 and documented that metastatic neoplasm had a significantly higher PASS value (score > 6) than the non-metastatic tumors. The main limitations of this scoring system are that it can be applied only to PCCs and that the morphological criteria evaluated for the score definition may be extremely variable with significant inter-observer and intra-observed variation, as shown by the following studies. In particular, Wu et al. found that those also reviewed by five multi-institutional pathologists with at least 10 years of experience in endocrine pathology, the assessment of PASS was remarkably variable, thus making its robustness and reproducibility difficult [60]. 

### 4.2. Grading System for Adrenal Pheochromocytoma and Paraganglioma (GAPP)

To define robust histopathological predictors of metastases development, a scoring system called grading system for adrenal pheochromocytoma and paraganglioma (GAPP) was developed by the Phaeochromocytoma Study Group in Japan based on the analysis of 163 neoplasms, including 40 metastatic tumors [61]. It is available for both PCCs and PGLs and includes morphological, immunohistochemical, and biochemical elements. The criteria of GAPP are histological pattern, cellularity, comedo-type necrosis, capsular/vascular invasion, Ki67-labelling index, and catecholamine type. All tumors were marked from 0 to 10 points and were classified as one of the three types: well-differentiated (0–2 points), moderately differentiated (3–6 points), and poorly differentiated (7–10 points), as shown in Table 3. The authors found a positive correlation between GAPP score and metastatic potential and a negative correlation with metastasis-free interval. Furthermore, a subsequent retrospective cohort study validated the predictive ability of GAPP and proposed a modified GAPP classification, a combination of some GAPP parameters with the loss of *SDHB* staining (M-GAPP) [62]. Despite some promising preliminary results [61,62,63], GAPP seemed inadequate to discriminate metastatic from non-aggressive cases in one recent study conducted on a series of MEN2A-associated PPCs [58]. 

Recently, Wachtel H. et al. [63] conducted a large retrospective cohort study on 143 patients affected by PPGLs to evaluate PASS and GAPP as metastatic predictors and their correlation with survival outcomes. They found that PASS was not related to metastases and documented significant interobserver variability; GAPP score had some predictive value for distant metastasis but not for local recurrence; strangely, poorly differentiated GAPP score neoplasms had an excellent prognosis.

### 4.3. Composite Pheochromocytoma/Paraganglioma Prognostic Score (COPPS)

To better predict the outcome of PPGLs, Pierre et al. [64] proposed a new composite prognostic score called COPPS (composite pheochromocytoma/paraganglioma prognostic score), on the basis of a retrospective study conducted on a mono-centric cohort of 147 cases of PPGLs. It is based on clinical and pathological features (tumor size, necrosis, and vascular invasion) and the losses of PS100 and *SDHB* immunostaining to predict the risk of metastasis, as shown in Table 4. An interesting result of this study also concerns the prognostic value of the loss of sustentacular cells, as suggested by other previous studies [38,65]. The authors showed that this score is able to correlate with progression-free survival (PFS) and metastatic behavior. Furthermore, it has both high sensitivity and specificity, but further studies are needed to confirm these preliminary results. Table 5 summarizes the main features of the presented histopathological-scoring systems.

## 5. Multivariable Prediction Models

Several studies aimed to assess the risk of recurrence in PPGLs by analyzing some of the parameters presented and discussed in the previous sections. Only a few of them, however, examined their independent predictive performance through multivariable predictive models [10,14,22,35,66]. 

Ayala-Ramirez et al. [10] conducted a large retrospective study of 371 cases of PPGLs to measure overall survival and disease-specific survival related to tumor size and location. The authors found that metastases were more commonly found in the cases of some of the tumor locations, such as the mediastinum and the infradiaphragmatic paraaortic areas, including the organ of Zuckerkandl. Patients with metastatic disease showed larger primary neoplasms, and tumor size was also associated with shorter overall survival. 

A large retrospective multicenter study conducted on 242 patients with PPGLs undergoing radical surgery in Piedmont by our group [14] showed that after a median follow-up of 73.9 months, 17.4% of patients had recurrence. Factor associated with recurrent disease were younger age at diagnosis, a positive family history of PPGLs, presence of specific mutations, larger tumors, and higher values of PASS for PCCs. In fact, the multivariate analysis confirmed that in PPGLs, genetic mutations, younger age, and larger tumor size were independently associated with recurrence risk; in PCCs higher tumor size, genetic mutations, younger age, and PASS value were associated with recurrence. Regarding metastatic disease, tumor size was the only predictor in PPGLs, whereas in PCCs PASS value was also related to the development of metastases. 

Cho et al. [22] conducted a retrospective cohort study comprising 333 PPGL patients and proposed an integrated risk score for recurrence prediction called ASES-score (or ASS-score, if excluding extra-adrenal localization). Each considered variable, i.e., age ≤ 35 years, tumor size ≥ 6.0 cm, extra-adrenal localization, and norepinephrine-secretory type were assigned 1 point (otherwise 0 points); these points were added to create the previously mentioned score. There was a significant difference in metastasis-free survival between patients with ASES-score ≥ 2 and <2. The negative predictive value of this system was 96.5% for a cut-off point of 2. The main limitation of this score, however, is that only clinical/biochemical features were considered, thus excluding the two most widely recognized predictors of recurrent and/or metastatic disease, genetic testing and histopathological data. 

Our group [66] also conducted a retrospective multicenter study on 177 PCC patients who underwent radical surgery. In this article, we proposed a multivariable continuous model for post-surgical PCC recurrence prediction, developed through a supervised regression approach by the integration of genetic, histopathologic, and clinical data. The variables finally included were age, tumor size, histopathological abnormalities, and genetic germline mutations in known susceptibility genes. The model was named the SGAP-model (size, genetic, age, and PASS). Despite some limitations, such as the sample size, the lack of genetic evaluation in all patients, the impossibility to separate patients with different genetic mutations, and the complex formula to estimate the outcome, it could represent a very useful tool for risk stratification if externally validated in a large cohort. To simplify this tool and allow an easier in clinical practice, we [67] reviewed the same patients’ data to create a discrete score through supervised regression and machine-learning techniques. We considered the same variables included in the SGAP-model, but to derive a simpler scoring system, continuous variables were dichotomized, using as cut points > 50 mm for tumor size, ≤35 years for age, and ≥3 for PASS. A novel prognostic score called the SGAP-score was thus created on an 8-point scale, by assigning 1 point for tumor size > 50 mm, 3 points for positive genetic testing, 1 point for age ≤ 35 years, and 3 points for PASS ≥ 3. Patients with a SGAP-score of 0–2 showed a virtually absent risk of recurrence; patients with a SGAP-score of 3–4 showed an intermediate risk profile; patients with a SGAP-score of 5–8 showed a markedly elevated risk of recurrence that exceeded 60% after 10 years. The proposed 3-class clustering, therefore, demonstrated a remarkable discriminative performance in the stratification of PCC-recurrence risk, which could be helpful if externally validated for a finer tailoring of post-surgical follow-up in radically operated PCC patients.

The comparison between the two clinical predictive-scoring systems is summarized in Table 6. 

## 6. Conclusions

**Figure 1:**
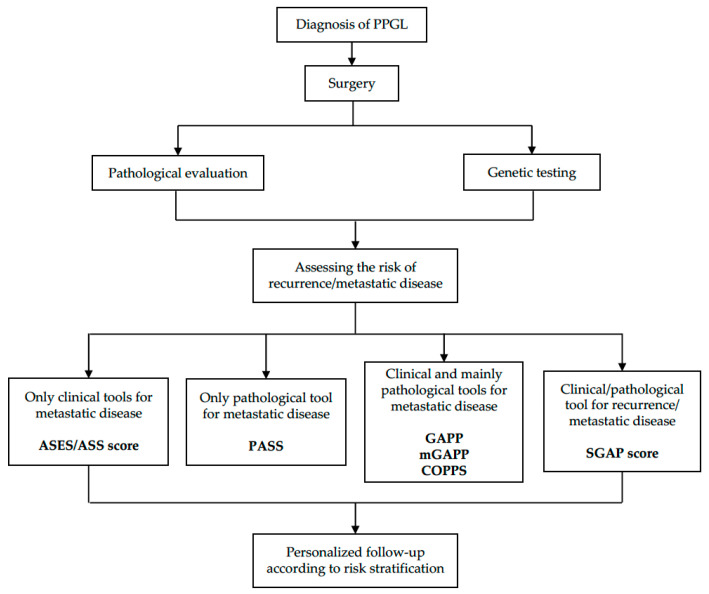
Flow-chart summarizing the main tools for the estimation of recurrence risk/metastatic potential and therefore, for defining the personalized follow-up of PPGL patients.

Clinical experience on this topic is difficult to achieve due to the low prevalence of PPGLs. Many clinically relevant questions remain so far unanswered. The appropriate duration of follow-up is still debated, as new events may be detected many years after the initial surgery. The same uncertainty holds with respect to the follow-up timing and intensity with a wide heterogeneity of more- or less-intensive schedules being proposed. Finally, the possibility to anticipate/discriminate the prediction of different recurrence types (i.e., new primary tumors, local relapse, or distant metastases) remains far from being reliably solved. The follow-up should be personalized, considering the recurrence/metastatic potential of single tumors/patients (Figure 1). Therefore, an accurate estimation of recurrence risk would be of fundamental importance in clinical practice, as it may allow clinicians to consider less-intensive schedules when the estimated recurrence risk is low, while suggesting a higher-intensity monitoring when the estimated recurrence risk is high. Although several variables have been found to be associated with PPGLs prognosis, either from a genetic, histopathological, or clinical point of view, to date there are still no validated multivariable models or scores recognized as reliable predictors of PPGL metastatic potential or recurrence risk [66] Some predictive tools have been recently proposed and appear to offer promising results, but they need to be externally validated on a larger scale. Therefore, a multidisciplinary and individualized approach to the treatment of patients affected by PPGLs is needed and international collaborative studies are required to further improve disease management and tailor patients’ treatments and follow-ups. 

## Figures and Tables

**Table 1:** Main studies on clinical predictors of metastatic disease/recurrence in PPGL. Abbreviations: PCC, pheochromocytoma; PGL, paraganglioma; PPGL, pheochromocytoma/paraganglioma; DA, dopamine; CgA, chromogranin A; NE, norepinephrine; E, epinephrine; VMA, vanillylmandelic acid; MN, metanephrine.

First Author, Year	Type of Study	Population	Patients	Outcomes	Clinical Predictors
John, 1999 [39]	Retrospective	PCC	86	Metastatic disease	Higher DA; extra-adrenal location; high tumor weight
Rao, 2000 [32]	Retrospective	PCC	27	Metastatic disease	Higher CgA; higher NE; lower E
Van der Harst, 2000 [26]	Retrospective	PCC	87	Metastatic disease	Higher DA; higher NE; lower ratio E/E + NE
Amar, 2005 [35]	Retrospective	PPGL	192	Recurrence	Younger age; familial disease;tumor site and size
Ayala-Ramirez, 2011 [10]	Retrospective	PPGL	371	Metastatic disease	Larger tumor size;extra-adrenal location
Park, 2011 [33]	Retrospective	PCC	152	Metastatic disease	Tumor > 5.5 cm; lower E, NE, VMA
Feng, 2011 [36]	Retrospective	PCC	136	Metastatic disease	Tumor > 5cm; multifocal and extra-adrenal tumors; higher MN
Eisenhofer, 2012 [28]	Retrospective	PPGL	365	Metastatic disease	Higher methoxytyramine; *SHDB* mutation; tumor > 5 cm;extra-adrenal location
De Wailly, 2012 [38]	Retrospective	PCC	53	Metastatic disease	Larger tumor size andhigher tumor weight
Press, 2014 [34]	Retrospective	PCC	135	Recurrence	Tumor > 5 cm
Kim, 2016 [23]	Retrospective	PPGL	223	Metastatic disease and/or recurrence	Younger age; germline mutations
Assadipour, 2017 [37]	Retrospective	PPGL	256	Metastatic disease and/or recurrence	*SDHB* mutation; tumor size
Hescot, 2019 [4]	Retrospective	PPGL	169	Metastatic disease	*SDHB* mutations
Parasiliti-Caprino, 2020 [14]	Retrospective	PPGL	242	Metastatic disease and/or recurrence	Genetic mutations; younger age; larger tumor size
Li, 2021 [21]	Retrospective	PPGL	249	Metastatic disease	Genetic mutations; lower E

**Table 2:** Pheochromocytoma of the adrenal gland scaled score (PASS). Abbreviations: HPF, high-power field.

Parameters	Score
Large nests or diffuse growth (>10% of tumor volume)	2
Central of confluent tumor necrosis	2
High cellularity	2
Cellular monotony	2
Tumor cell spindling	2
Mitotic figures > 3/10 HPF	2
Atypical mitotic figure(s)	2
Extension into adipose tissue	2
Vascular invasion	1
Capsular invasion	1
Profound nuclear pleomorphism	1
Nuclear hyperchromasia	1
Total maximum	20

**Table 3:** Grading of adrenal pheochromocytoma and paraganglioma (GAPP). Abbreviations: E, epinephrine; NE, norepinephrine; DA, dopamine; U, number of tumor cells in a square of a 10 mm micrometer observed under high-power magnification (×400).

Parameters	Score
Histological pattern Zellballen Large and irregular cell nest Pseudorosette	0 1 1
Cellularity Low (<150 cells/U) Moderate (150–250 cells/U) High (>250 cells/U)	0 1 2
Comedo necrosis Absence Presence	0 2
Vascular or capsular invasion Absence Presence	0 1
Ki67-labelling index (%) <1 1–3 >3	0 1 2
Catecholamine type E or E + NE NE or NE + DA Non-functioning	0 1 0
Total maximum	10

**Table 4:** Composite pheochromocytoma/paraganglioma prognostic score (COPPS). Abbreviations: SDHB, succinate dehydrogenase complex subunit B.

Parameters	Score
Focal or confluent necrosis	5
PS100 loss	2
Vascular invasion	1
SDHB loss	1
Tumor size > 7 cm	1
Total maximum	10

**Table 5:** Comparisons between PASS, GAPP and COPPS. Abbreviations: PCC, pheochromocytoma; PPGL, pheochromocytoma/paraganglioma.

	PASS	GAPP	COPPS
Metastatic risk stratification	≥4: high metastatic risk	0–2: well differentiated 3–6: moderately differentiated 7–10: poorly differentiated	≥3 high metastatic risk
Application	PCC	PPGL	PPGL
Parameters	Histological	Histological/clinical	Histological/clinical/molecular

**Table 6:** Comparison between SGAP-score and ASES/ASS-score. Abbreviations: NE, norepinephrine; E, epinephrine.

	SGAP-Score	ASES/ASS-Score
Variables of scoring system	*Tumor size* >5 cm: 1 ≤5 cm: 0 *Age* ≤35: 1 >35: 0 *Genetic testing* Positive: 3 Negative: 0 *PASS* ≥3: 3 <3: 0	*Tumor size* ≥6 cm: 1 <6 cm: 0 *Age* ≤35: 1 >35: 0 *Tumor location* Extra-adrenal: 1 Adrenal: 0 *Secretory profile* NE-secretory type: 1 E-secretory type: 0
Risk stratification	Low risk: 0–2 Intermediate risk: 3–4 High risk: 5–8	Poor prognosis: ≥2 Better prognosis: <2
Outcomes	Recurrence of any type	Metastatic disease
Parameters	Clinical, genetic, and histopathological	Only clinical

## Data Availability

Not applicable.
